# CD200-CD200R1 inhibitory signaling prevents spontaneous bacterial infection and promotes resolution of neuroinflammation and recovery after stroke

**DOI:** 10.1186/s12974-019-1426-3

**Published:** 2019-02-18

**Authors:** Rodney M. Ritzel, Abdullah Al Mamun, Joshua Crapser, Rajkumar Verma, Anita R. Patel, Brittany E. Knight, Nia Harris, Nickolas Mancini, Meaghan Roy-O’Reilly, Bhanu Priya Ganesh, Fudong Liu, Louise D. McCullough

**Affiliations:** 1 Department of Anesthesiology, Center for Shock, Trauma and Anesthesiology Research (STAR) Center, University of Maryland School of Medicine, Baltimore, MD USA; 2 Department of Neurology, McGovern Medical School, University of Texas Health Science Center, 6431 Fannin Street, Houston, TX 77370 USA; 3 Neuroscience Department, University of Connecticut Health Center, 263 Farmington Avenue, Farmington, CT 06030 USA

**Keywords:** CD200R1, Microglia, Ischemic stroke, Immunosuppression

## Abstract

**Background:**

Ischemic stroke results in a robust inflammatory response within the central nervous system. As the immune-inhibitory CD200-CD200 receptor 1 (CD200R1) signaling axis is a known regulator of immune homeostasis, we hypothesized that it may play a role in post-stroke immune suppression after stroke.

**Methods:**

In this study, we investigated the role of CD200R1-mediated signaling in stroke using CD200 receptor 1-deficient mice. Mice were subjected to a 60-min middle cerebral artery occlusion and evaluated at days 3 and 7, representing the respective peak and early resolution stages of neuroinflammation in this model of ischemic stroke. Infarct size and behavioral deficits were assessed at both time points. Central and peripheral cellular immune responses were measured using flow cytometry. Bacterial colonization was determined in lung tissue homogenates both after acute stroke and in an LPS model of systemic inflammation.

**Results:**

In wild-type (WT) animals, CD200R1 was expressed on infiltrating monocytes and lymphocytes after stroke but was absent on microglia. Early after ischemia (72 h), CD200R1-knockout (KO) mice had significantly poorer survival rates and an enhanced susceptibility to spontaneous bacterial colonization of the respiratory tract compared to wild-type (WT) controls, despite no difference in infarct or neurological deficits. While the CNS inflammation was resolved by day 7 post-stroke in WT mice, brain-resident microglia and monocyte activation persisted in CD200R1-KO mice, accompanied by a delayed, augmented lymphocyte response. At this time point, CD200R1-KO mice displayed greater weight loss, more severe neurological deficits, and impaired motor function compared to WT. Systemically, CD200R1-KO mice exhibited signs of persistent infection including lymphopenia, T cell activation and memory conversion, and narrowing of the TCR repertoire. These findings were confirmed in a second model of acute neuroinflammation induced by systemic endotoxin challenge.

**Conclusion:**

This study defines an essential role of CD200-CD200R1 signaling in stroke. Loss of CD200R1 led to high mortality, increased rates of post-stroke infection, and enhanced entry of peripheral leukocytes into the brain after ischemia, with no increase in infarct size. This suggests that the loss of CD200 receptor leads to enhanced peripheral inflammation that is triggered by brain injury.

**Electronic supplementary material:**

The online version of this article (10.1186/s12974-019-1426-3) contains supplementary material, which is available to authorized users.

## Background

Ischemic stroke is currently the fifth leading cause of death and the leading cause of long-term disability in the USA [1]. Stroke-induced inflammation is believed to contribute to neuronal damage, injury progression, and clinical outcome [2]. In particular, phagocytic microglia and macrophages of the innate immune system have been shown to play pivotal roles in the brain’s response to injury. Microglia, the resident immune cells of the CNS; and macrophages; and their peripheral counterparts primarily serve to maintain immune homeostasis via tightly regulated pro- and anti-inflammatory signaling pathways [3]. However, in a pathological setting like ischemic stroke, this signaling can become overactive or dysregulated, exacerbating neuronal cell death and loss of function [4].

Among the homeostatic mechanisms that regulate the activation state of microglia/macrophages are a group of membrane-associated glycoproteins known as immune inhibitory receptors, which are responsible for regulating CNS inflammation via neuronal-glial interactions. Emerging data has shown that the interaction of CD200R1, an inhibitory immune receptor expressed on myeloid cells, with the CD200 ligand expressed on neurons leads to downstream inhibition of pro-inflammatory pathways, maintaining microglia in an inactive, resting state [5, 6]. Following tissue injury, these interactions between neurons and microglia are disrupted, lifting the “brake” on inflammation and enabling a pro-inflammatory response. While it is likely that CD200-CD200R1 signaling may be disrupted after stroke [7–11], the functional contribution of the CD200-CD200R1 immune-inhibitory signaling axis in stroke is not currently known.

Interestingly, ischemic stroke results in a dual-edged peripheral inflammatory response, characterized by an early increase in pro-inflammatory cytokine production followed by a state of profound immunosuppression [12]. This results in an increased susceptibility to pulmonary and urinary tract infections, affecting approximately 20% of all stroke patients and significantly increasing mortality rates [13, 14]. However, the role of immune inhibitory molecules like CD200R1 in the peripheral inflammatory response and susceptibility to infection after brain injury remains largely unexplored.

In this study, we investigated the role of CD200-CD200R1 signaling on experimental stroke outcome and post-stroke infection risk. CD200R1-knockout (KO) and wild-type (WT) littermate control mice were subjected to 60-min transient middle cerebral artery occlusion (tMCAO) and evaluated up to 1 week for post-acute changes in microglia proliferation, monocyte infiltration, and behavioral deficits. We demonstrate that CD200R1 inhibitory signaling functions as a critical regulator of the peripheral immune response after brain injury, impacting survival and the susceptibility to post-stroke infection. In light of this, the CD200R1 axis may represent a potential therapeutic target for the treatment of ischemic stroke and other sterile brain injuries, via the suppression of deleterious leukocyte activation and amelioration of stroke-induced lymphopenia.

## Methods

### Mice/animals

CD200R1^+/−^ mice (bred on a C57BL/6J background) were generously provided by Professor R. Gorczynski (Toronto, Canada) [15]. Heterozygous CD200R1^+/−^ mice were bred to obtain CD200R1^−/−^ mutants and CD200R1^+/+^ wild-type littermates. Young adult male mice (12–14 weeks of age) were pair-housed on sawdust bedding in a pathogen-free facility (light cycle 12/12 h light/dark). All animals had access to chow and water ad libitum.

### Ischemic stroke model

Cerebral ischemia was induced by 60 min of transient MCAO under isoflurane anesthesia as previously described [16]. Rectal temperatures were maintained at approximately 37 °C during surgery and ischemia with an automated temperature control feedback system. Briefly, a midline ventral neck incision was made, and unilateral MCAO was performed by inserting a 6.0 Doccol monofilament (Doccol Corp, Redland, CA) into the right internal carotid artery 6 mm from the internal carotid/pterygopalatine artery bifurcation via an external carotid artery stump. Sham-operated animals underwent the same surgical procedure, but the suture was not advanced into the internal carotid artery. All animals were randomly assigned to stroke/sham surgery, and analysis was performed blinded to the surgical conditions.

### Behavioral testing

Body weight was monitored daily. Neurological deficits were assessed by the Bederson score system from 0 (no deficit) to 4 (severe deficit), with minor modifications [17]. Mice underwent a variety of behavioral testing, including nesting activity (overnight), neurological scoring, static rod, and rotarod. All the animals were tested on each behavioral task 3 days prior to surgery, to establish a baseline, and again on the day of sacrifice. All mice included in this study did not demonstrate any behavioral deficits at baseline. All testing was performed at a fixed time (09:00 am–12:00 am) by an investigator blinded to the genotype and surgical condition.

### Static rod

Balance and coordination were assessed using the static rod test, a well-validated assessment of motor balance [18, 19]. In this test, a wooden rod of 60 cm in length and 2.8 cm in diameter was used as previously described [20], with minor modification. Briefly, the rod was fixed by a G-clamp to a laboratory table such that the rod horizontally protruded into space 60 cm above a cotton-padded floor. Mice were placed at the free end of a fixed horizontal rod 60 cm above a cotton-padded floor facing away from the home table (nose tip one head’s length from the edge) and were allowed a maximum period of 180 s to turn 180°. The time taken to achieve this orientation was scored for each mouse and taken as an average of three trials. The number of falls and successful turns was also tallied for each trial, and the percentage was calculated.

### Rotarod

Mice were placed on a rotating cylindrical rod accelerating from 2 to 20 rpm, over a span of 5 min [21, 22]. Subjects were tested over four trials at baseline and on day 7 post-stroke, with a 5-min break between trials. The latency of the subject to fall from the rotating rod was recorded for each trial (in seconds), and the average latency was used for further analysis. No differences were found between groups at baseline.

### Nesting activity

Two hours prior to the onset of the dark phase, mice were separated into individual cages and supplied with a ~ 5 cm × 5 cm pressed cotton square. Nesting activity was assessed as described previously [23, 24], with minor modifications. In brief, after mice had habituated for 15 min in a novel empty cage, the cotton pad was weighed and placed into a randomized cage corner. The next morning (approximately 12 h later), the cages were inspected for nest construction. Any loose, shredded material was gently brushed off the intact nesting pad. Nesting activity was calculated as follows: [1 − (post-pad wt. (g)/pre-pad wt. (g))] × 100 = % nested material.

### Terminal histopathology

All animals were sacrificed at 72 h or 7 days after stroke via avertin overdose (i.p). For the 72-h cohorts, the brains were collected and hardened at − 20 °C for 5 min, cut into five 2-mm coronal sections, and stained with 1.5% 2,3,5-triphenyltetrazolium chloride (TTC) for 8 min at 37 °C. The slices were formalin-fixed (4%) and infarct volumes analyzed using Sigma Scan Pro software as previously described [25]. Final infarct volumes were presented as a percentage of contralateral structures, with correction for edema. A separate cohort of animals was sacrificed at 7 days for sub-acute infarct analysis and immunohistochemistry. After transcardial perfusion with cold PBS followed by 4% paraformaldehyde, the brains were removed and fixed for 24 h in 4% paraformaldehyde (PFA) before being placed into a cryoprotectant solution (30% sucrose). The brains were cut into 40-μm free-floating sections on a freezing microtome, and every eighth slice was stained by Cresyl violet (CV) staining to evaluate ischemic cell damage. The images were digitalized, and cerebral atrophy was analyzed using computer software (Sigma scan Pro5) as previously described [26, 27].

### India ink vascular staining

WT and KO mice were anesthetized (isoflurane) and perfused with PBS followed by 4% paraformaldehyde and India ink (50% India ink, 5% FeSO4 in PBS). The brains were harvested and visualized using a digital camera after brief refrigeration (Pansonic HC V770).

### Immunohistochemistry

Immunohistochemical staining of fixed-frozen sections (40 μm thickness) was performed as described previously [28]. Briefly, the brain slices were mounted onto gelatin-coated slides, and primary antibody (rabbit anti-Iba1, Wako, 1:250) was added overnight. Secondary antibodies (1:1000) with corresponding conjugates and 4′,6-diamidino-2-phenylindole and dihydrochloride (DAPI, 1:1000, Invitrogen, Carlsbad, CA) were applied. Images were analyzed with a Zeiss Axiovert 200M microscope (Carl Zeiss, Oberkochen, Germany) and ImageJ software (NIH).

### Tissue processing for flow cytometry

Mice were euthanized and transcardially perfused with 60 mL cold sterile PBS, and the brains were harvested. The blood was drawn with heparin-coated needles and subjected to three rounds of 10 min red blood cell lysis with Tris ammonium chloride on ice. The ipsilateral hemispheres were placed in complete RPMI 1640 (Lonza) medium and mechanically and enzymatically digested in collagenase/dispase (1 mg/mL) and DNAse (10 mg/mL; both Roche Diagnostics) for 1 h at 37 °C. The cell suspension was filtered through a 70-μm filter and placed into a 70%/30% Percoll gradient. Following centrifugation, leukocytes were harvested from the interphase portion of the gradient. Cells were washed and blocked with mouse Fc Block (CD16/CD32, eBioscience) prior to staining with primary antibody-conjugated fluorophores (eBioscience): CD45-eF450 (clone: 30-F11), CD11b-APCeF780 (clone: M1/70), CD3e-APC (clone: 17A2), Ly6C-PerCP-Cy5.5 (clone: HK1.4), and Ly6G-PE (clone: 1A8). For live/dead discrimination, a fixable viability dye, carboxylic acid succinimidyl ester (CASE-AF350, Invitrogen), was diluted at 1:300 from a working stock of 0.3 mg/mL. Data was acquired on a LSRII using FACsDiva 6.0 (BD Biosciences) and analyzed using FlowJo (Treestar Inc.). A gating strategy was designed as previously described [29]. Resident microglia were identified as the CD45^int^CD11b^+^Ly6C^−^ population, whereas bone marrow-derived leukocytes were identified as CD45^hi^. Cell type-matched fluorescence minus one (FMO) controls were used to determine the positive gating for each antibody. The following antibodies were also used in this study: CD11a-APC (clone: M17/4, Biolegend), CD44-FITC (clone: IM7, eBioscience), CD69-APC (clone: H1.2F3, eBioscience), CD200R1-FITC (clone: OX110, Biolegend), CD19-AF700 (clone: 6D5, eBioscience), MHCII-Bv510 (clone: M5/114.15.2, Biolegend), NKp46-PerCPeF710 (clone: 29A1.4, eBioscience), and CD49b-PE (clone: DX5, Biolegend).

### TCRvβ usage

We used a mouse vβ TCR screening panel (BD Pharmingen) according to the manufacturer’s instructions. Briefly, blood leukocytes from each sample were divided into 15 separate FACS tubes and stained for the antibody cocktail including 1 of 15 respective TCRvβ FITC-conjugated monoclonal antibodies [30].

### Phagocytosis assay

Prior to blocking, fluorescent latex beads (Fluoresbrite Yellow Green (YG) carboxylate microspheres; 1um diameter; Polysciences) were added to the isolated microglia ex vivo in a final dilution of 1:100 as described [29, 31]. After 1 h incubation at 37 °C with periodic agitation, the cells were washed three times with FACS buffer, resuspended in FACS buffer, stained for surface markers, and fixed in PFA.

### Intracellular cytokine production

For intracellular cytokine staining, a stock solution of brefeldin A (Sigma) was prepared at 20 mg/mL in DMSO and diluted with PBS to obtain a working solution of 0.5 mg/mL. Mice were euthanized 12 h after intravenous injection of brefeldin A (250 μL). Leukocytes were collected as described above, and 1 μL of GolgiPlug containing brefeldin A (BD Biosciences) was added to 800 μL complete RPMI. Cells were resuspended in Fc Block, stained for surface antigens, and washed in 100 μL of fixation/permeabilization solution (BD Biosciences) for 20 min. Cells were then washed twice in 300 μL permeabilization/wash buffer (BD Biosciences) and resuspended in an intracellular antibody cocktail containing TNF-PE-Cy7 (clone: MP6-XT22, eBioscience) and IL-1β-FITC (clone: NJTEN3, eBioscience), followed by fixation.

### Endotoxin challenge model

Mice were injected with lipopolysaccharide (LPS) endotoxin derived from *Escherichia coli* O114:B4 (strain O111:B4; Sigma-Aldrich, St. Louis, MO, USA). LPS was dissolved in phosphate-buffered saline (PBS, pH 7.4) at a concentration of 1 mg/mL. A frozen aliquot of the LPS was prepared in normal saline on the day of use. To generate peripheral inflammation, animals were given a single i.p. injection of LPS at 5 mg/kg.

### Sickness behavior

Sickness behavior is normally a temporary state characterized by adaptive behavioral- and neuroimmune-specific changes orchestrated by the host to fight the invading pathogens [32, 33]. Typical symptoms of sickness behavior are reduced posture movement (scored as follows: 0 = normal behavior, 1 = hunched but still avoid moving, 2 = hunched and not moving), eye squinting (a symptom of discomfort scored as follows: 0 = no eye squinting, 1 = moderate eye squinting in both eye, 2 = severe eye squinting in both eye), hair striking (scored as follows: 0 = normal behavior, 1 = hair is only partially sticking up, 2 = hair sticking up on the whole back), prostration touch response (scored as follows: 0 = a normal mouse runs away and tries to avoid touch, 1 = moderate response, 2 = animal was completely unresponsive), and weight loss.

### Measurement of brain cytokine and hemoglobin concentrations

The ipsilateral brain was homogenized using a Dounce homogenizer in ten volumes of NP40 cell lysis buffer (FNN0021, Thermo Fisher Scientific, USA) supplemented with 1 mM phenylmethylsulphonyl fluoride (PMSF) and a protease inhibitor cocktail (Sigma-Aldrich). All steps were carried out at 4 °C. The homogenate was centrifuged initially at 700×*g* for 5 min to eliminate unruptured cells and debris and then further centrifuged at 12,500*g* for 20 min. The supernatant was used to measure cytokine levels by ELISA. Tumor necrosis factor-alpha (TNF-α) and IL-1β levels were measured by commercially available specific quantitative multiplex ELISA kits according to the manufacturer’s instructions (# M60000007A, Bio-Rad Laboratories, Hercules, CA). Hemoglobin levels were measured by commercially available colorimetric determination of total hemoglobin according to the manufacturer’s instructions (# DIHB-250, BioAssay Systems, Hayward, CA).

### Complete blood count assay

Complete blood count (CBC) was performed by using IDEXX ProCyte Dx Hematology Analyzer according to the manufacturer’s instructions (IDEXX, Westbrook, Maine, USA). Briefly, approximately 300 μL of whole blood from each animal was diluted in 100 μL of 0.5 M EDTA (Sigma), then diluted samples were placed in an automated ProCyte Dx Hematology Analyzer.

### CD200 ELISA

Protein concentrations were assayed using a mouse CD200 PicoKine ELISA kit (Boster Bio, Pleasanton, CA). Plasma samples and 100 μg of whole cell lysate brain protein were plated in triplicate for each sample and assayed according to the manufacturer’s instructions using a microplate reader (EnSpire 2300 Multilabel Reader, Perkin Elmer). CD200 protein concentration was quantitatively determined by measuring the optical density absorbance at 450 nm.

### Serum LPS-binding protein assay

LPS-binding protein (LBP) in serum was measured by commercially available specific quantitative sandwich ELISA kits according to the manufacturer’s instructions (# KA0449, Abnova, Walnut, CA, USA).

### Lung CFU counts

After sacrifice, both the left and right lungs were harvested from mice under sterile conditions and maintained at 4 °C. Half of the tissue from both lungs was homogenized in sterile 1× PBS, and the homogenate was allowed to sit for 1 min at room temperature. Tenfold serial dilutions were obtained from the lung homogenate, ranging from 10^−1^ to 10^−10^. Each dilution was plated on a blood agar plate and incubated under anoxic conditions (Coy Laboratory Products) at 37 °C chamber overnight [34, 35]. Positive colonies were recorded, and colony-forming units (CFU) were calculated per gram of lung tissue (wet weight).

### Statistical analyses

Data from individual experiments are presented as mean ± SEM and assessed by Student *t* test or one-/two-way ANOVA with Tukey post-hoc test for multiple comparisons and Hold-Sidak test for paired comparisons (GraphPad Prism Software Inc., San Diego, CA, USA). Kaplan-Meier survival curves were compared using Cox-Mantel analysis to determine statistical significance between the groups. A significance was set at *p* ≤ 0.05. The neurological deficit scores and sickness behavioral scores were analyzed using the Mann-Whitney *U* test. Data obtained from behavioral testing and flow cytometry experiments are representative of two biological replicates using animals from different litters. All the studies were performed by investigators blinded to the genotypes of the mice.

## Results

### CD200R1-deficient mice have higher mortality associated with exacerbated microgliosis and monocyte infiltration at 72 h after stroke independent of infarct volume

**Fig. 1:**
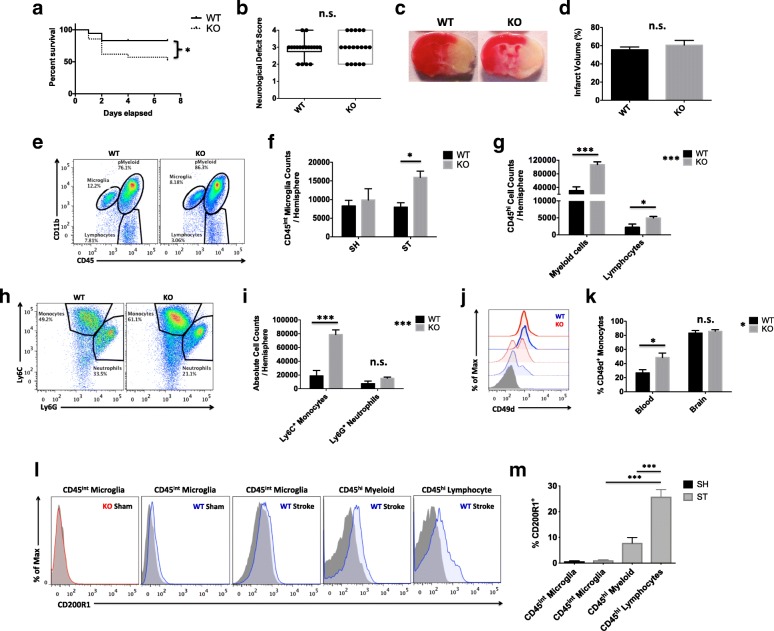
CD200R1-KO mice have greater mortality associated with exacerbated microgliosis and monocyte infiltration independent of infarct size at 72 h after ischemic stroke. A Kaplan-Meier survival curve shows the extent of mortality in each group of a cohort following 60 min of MCAO (**a**; *N* = 18–21/group). Neurological deficit scores were assessed for each group at 72 h (**b**; *N* = 18/group). Representative images of 2,3,5-triphenyltetrazolium chloride (TTC)-stained coronal brain sections for CD200R1-knockout (KO) and CD200R1-wild-type (WT) control littermates at 72 h after 60 min of middle cerebral artery occlusion (MCAO) (**c**). Infarct volumes were measured for the total ipsilateral hemispheric area relative to the contralateral side (**d**; *N* = 9/group). Representative dot plots depict the extent of CD45^int^CD11b^+^ microgliosis and CD45^hi^CD11b^+^ myeloid cell infiltration in the ischemic brain of each group (**e**). The absolute number of microglia (CD45^int^CD11b^+^Ly6C^−^) was quantified at 72 h (**f**; *N* = 7/group). The absolute number total infiltrating myeloid cells (CD45^hi^CD11b^+^) and putative lymphocytes (CD45^hi^CD11b^−^) was quantified (**g**). A dot plot shows the identification of specific myeloid subsets in the ischemic brain (**h**). The number of monocytes (CD45^hi^CD11b^+^Ly6C^+^Ly6G^−^) and neutrophils (CD45^hi^CD11b^+^Ly6C^+^Ly6G^+^) was compared between genotypes (**i**). The percentage of CD49d-positive monocytes in the blood and brain of stroke-injured mice is shown (**j**) by histogram (CD200R1-WT = blue, CD200R1-KO = red, blood = dotted line, brain = solid line, FMO = shaded gray). Quantification of these data shows greater α4-integrin upregulation on circulating monocytes in CD200R1-KO mice (**k**). Representative histograms illustrate the relative level of CD200R1 expression on CD45^int^CD11b^+^ microglia, CD45^hi^CD11b^+^ myeloid cells, and CD45^hi^CD11b^−^ lymphocytes in the brain at 72 h after stroke (**l**; KO = red, WT = blue). Quantification of these data is shown (**m**; *N* = 7–15/group). Asterisks adjacent to group labels designate an effect of genotype by one-way ANOVA. FMO controls were used to determine positive gating. Error bars show mean SEM. SH sham, ST stroke, KO knockout, WT wild-type, SEM standard error of mean. *p < 0.05; **p < 0.01; ***p < 0.001

To begin, we examined whether CD200R1 deficiency altered acute outcomes following experimental ischemic stroke. Despite significantly higher mortality rates in CD200R1-KO mice within the first week of injury (*p* < 0.05), no difference between neurological deficits scores was found at day 3 after MCAO (Fig. 1a, b). Consistent with this finding, infarct analysis revealed no statistical difference between the groups (Fig. 1c, d). However, both the central and peripheral neuroinflammatory response was augmented in CD200R1-KO mice as measured by brain flow cytometry. Significantly greater numbers of microglia were seen in CD200R1-KO mice compared to WT at 72 h post-stroke, despite no differences in microglia counts at baseline (Fig. 1e, f; *p* ≤ 0.05). In addition, infiltrating peripheral lymphocyte and myeloid cell counts were significantly higher in the CD200R1-KO brains at 72 h compared to WT (Fig. 1g). Further resolution of myeloid subsets revealed that monocyte numbers were approximately four times greater in the ischemic hemisphere of CD200R1-KO mice compared to WT controls, with no significant difference in neutrophil counts (Fig. 1h, i; *p* ≤ 0.001). Further surface phenotyping showed that the integrin adhesion molecule CD49d is significantly upregulated on circulating monocytes deficient for CD200R1, suggesting that increased leukocyte-endothelial contact might facilitate enhanced entry of monocytes into the ischemic brain of CD200R1-KO mice (Fig. 1i–k). Next, we examined the expression of CD200R1 protein on brain-resident microglia and peripheral infiltrating immune cells 72 h after experimental ischemic stroke. Interestingly, the surface expression of CD200R1 protein was nearly absent on microglia in healthy and ischemic brains (Fig. 1l, m). Modest expression was found on brain-infiltrating myeloid cells, and comparably higher expression was found on the infiltrating lymphocyte population (*p* ≤ 0.001). No change in CD200 concentration was found in the brain or plasma after stroke in WT mice (Additional file 1: Figure S1). These findings suggest that the increased mortality seen in CD200R1-KO mice may be mediated by an aggravated inflammatory response independent of infarct size.

### Myeloid functions are differentially affected in the ischemic brain of CD200R1-KO mice

**Fig. 2:**
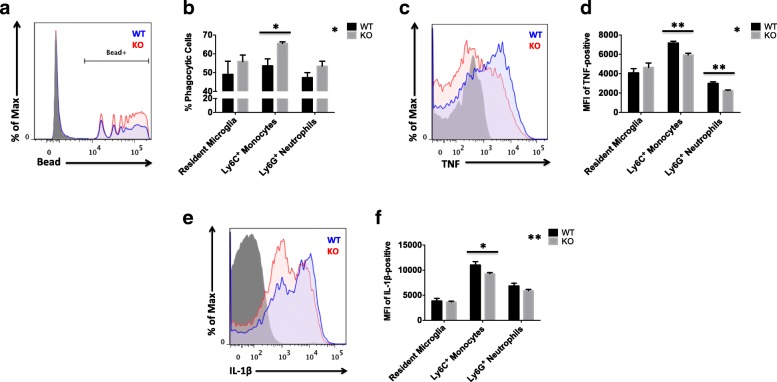
Lack of CD200R1-mediated immune inhibitory signaling differentially affects myeloid cell functions in the ischemic brain. Representative histograms illustrate the functional differences in phagocytic activity (**a**) and TNF-α (**b**) and IL-1β (**c**) production between infiltrating monocytes of CD200R1-KO (red) and WT littermate controls (blue) at 72 h after ischemic stroke. Cell-specific FMO controls were used to determine positive gating (shaded gray). Results from the ex vivo phagocytosis bead assay were quantified for resident microglia (CD45^int^CD11b^+^Ly6C^−^) and brain-infiltrating monocytes (CD45^hi^CD11b^+^Ly6C^+^Ly6G^−^) and neutrophils (CD45^hi^CD11b^+^Ly6C^+^Ly6G^+^) in each group (**b**; *N* = 6/group). The mean fluorescence intensities (MFI) of TNF-positive (**d**) and IL-1β-positive (**f**) myeloid cells were measured to assess differences in the relative protein expression level of these subsets between groups (*N* = 6 mice/genotype/surgery group). Asterisks adjacent to group labels designate an effect of genotype by two-way ANOVA. KO knockout, WT wild-type, SEM standard error of mean. *p < 0.05; **p < 0.01

As our initial experiments demonstrated that CD200R1 deficiency augments myeloid infiltration into the brain, we next examined the effect of CD200R1 loss on myeloid cell phenotype and function. Ex vivo assays were performed on freshly isolated leukocytes from the ischemic brain at 72 h after MCAO. Given the importance of clearing cellular debris following ischemic tissue damage, we first evaluated the level of phagocytic activity by each myeloid subset. Our data demonstrate a significant increase in the percentage of CD200R1-KO monocytes with enhanced phagocytic activity compared to their WT counterparts. No differences in microglia or neutrophil phagocytosis were seen between the groups (Fig. 2a, b; *p* ≤ 0.05). As heightened phagocytic activity is regarded as beneficial and is generally associated with an attenuated cytokine response, we next performed intracellular staining in these cells to examine the production of the pro-inflammatory cytokines TNF-α and IL-1β in response to ischemic brain injury. The relative protein expression levels of TNF-α and IL-1β were significantly lower in CD200R1-deficient monocytes after stroke compared to WT controls (Fig. 2c–f; *p* ≤ 0.01 and *p* ≤ 0.05, respectively). Although no differences were seen in microglia, CD200R1-deficient neutrophils expressed lower levels of TNF-α relative to their WT counterparts (Fig. 2d; *p* ≤ 0.01). Collectively, these data indicate that monocyte function is uniquely affected by CD200R1 deletion.

### CD200R1-KO mice have worse functional outcomes and behavioral deficits at day 7 after stroke, independent of neuronal injury

**Fig. 3:**
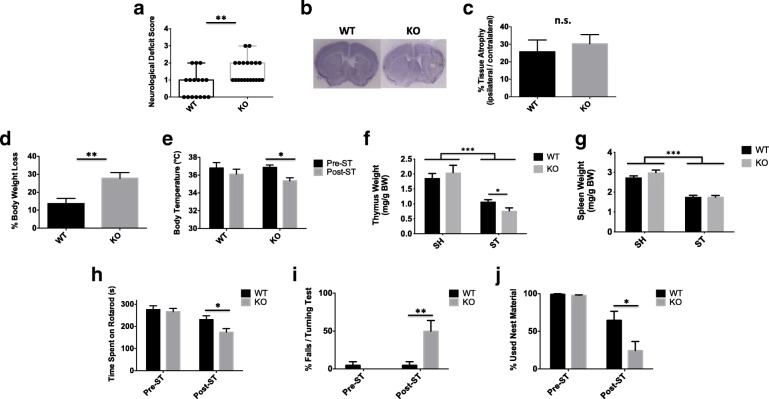
CD200R1-KO mice exhibit significantly worse functional outcomes at 1 week after stroke. Neurological deficit scores were assessed for each group at 7 days (**a**; *N* = 16–20/group). Representative images of Cresyl violet-stained coronal brain sections for CD200R1-KO and WT control littermates at 7 days after 60 min of middle cerebral artery occlusion (**b**). Tissue atrophy was measured for the total ipsilateral hemispheric area relative to the contralateral side (**c**; *N* = 8/group). The percentage of body weight loss is shown (**d**). Core body temperatures were measured (**e**), and spleen (**g**) and thymus (**f**) weights were normalized to body weight at 7 days (respectively *N* = 5 SH/group and *N* = 12–14 ST/group). The latency to fall on an accelerated rotarod device was recorded (**h**). CD200R1-KO mice displayed a significantly greater percentage of falls in a 180° turning test compared to WT controls (**i**). Nesting behavior was measured at day 7, and the percentages of used nest material are shown (**j**). For all behavior experiments, *N* = 12–14 mice per group. Error bars show mean SEM. Asterisks adjacent to group labels designate an effect of stroke by two-way ANOVA. KO knockout, WT wild-type, g grams, s seconds, BW body weight, SH sham, ST stroke, SEM standard error of mean. *p < 0.05; **p < 0.01

In order to test the temporal impact of CD200R1 signaling, we next examined the effects of CD200R1 deficiency on stroke outcomes at day 7 in addition to day 3. Interestingly, CD200R1-KO mice displayed significantly worse neurological deficit scores at day 7 compared to controls (Fig. 3a; *p* = 0.003), a detrimental effect of CD200R1 deletion not seen at day 3 (Fig. 1b). No significant difference was seen in tissue atrophy between groups by Cresyl violet staining (Fig. 3b, c). Notably, CD200R1-KO mice exhibited a dramatic decrease in body weight following stroke, nearly twice that of WT controls (Fig. 3d; *p* = 0.003), which was accompanied by significant dysregulation in body temperature (Fig. 3e; *p* ≤ 0.05). While, thymic atrophy persisted to a greater degree in CD200R1-KO mice at day 7 (Fig. 3f, *p* ≤ 0.05), no differences in splenic atrophy were found between the groups (Fig. 3g).

Next, to see whether these poor physiological outcomes were associated with deficits in functional recovery, we ran a battery of behavioral tests to measure sensorimotor deficits and anxiety/depressive features after stroke. Importantly, no differences were seen between genotypes at baseline. However, CD200R1-KO mice showed significant impairment in their ability to remain stable on an accelerating cylinder rod, having a shorter latency to fall (Fig. 3h, *p* ≤ 0.05). Similarly, we found that CD200R1-KO mice failed to successfully orient themselves without falling in approximately half of the trials on a static bar test (Fig. 3i, *p* ≤ 0.01). Lastly, we assessed for deficits in the ability to perform normal daily activities such as nest construction. CD200R1-KO mice showed a significant reduction in nesting activity 1 week after stroke as compared to WT controls (Fig. 3j, *p* ≤ 0.05). Collectively, this data implies that immune-inhibitory interactions in the CNS are not only critical for survival after stroke, but also for the recovery of motor processes involved in balance and coordination as well as the capacity to perform normal daily tasks.

### CD200-CD200R1 signaling promotes the resolution of leukocyte infiltration into the ischemic brain, resulting in improved neurological recovery

**Fig. 4:**
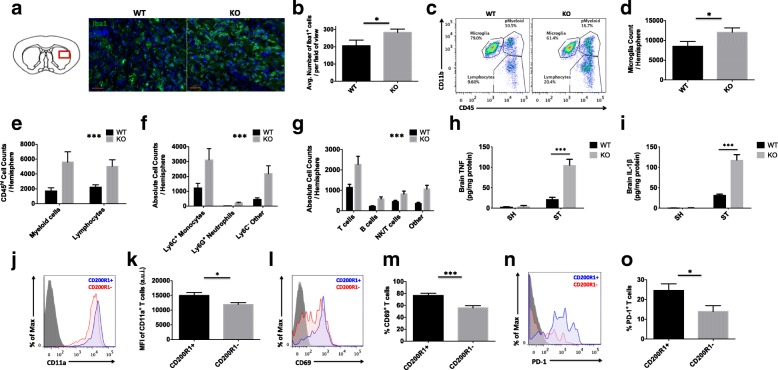
CD200R1-deficient mice demonstrate an inability to resolve neuroinflammation 1 week after ischemia. The inset of the diagram illustrates the striatal region bordering the penumbral zone from which the following representative histological sections were taken (**a**; scale bar = 50 μm). Sections were stained with the microglia/macrophage marker, anti-ionized calcium-binding adaptor molecule 1 (Iba1; in green), and the nuclear/DNA marker 4′,6-diamidino-2-phenylindole (DAPI; in blue). The average number of co-labeled Iba1^+^ microglia/macrophages in the region of interest was quantified for all sections (**b**; *N* = 8/group). Representative dot plots show greater numbers of CD45^+^CD11b^+^ and CD45^hi^CD11b^−^ leukocytes in the ischemic hemisphere of CD200R1-KO mice compared to WT controls at 7 days (**c**). The absolute number of CD45^int^CD11b^+^Ly6C^−^ microglia (**d**) and infiltrating CD45^hi^ myeloid and lymphocyte populations (**e**) was quantified (*N* = 13/group). A more detailed phenotypic analysis was performed to identify the specific myeloid (monocytes: CD45^hi^CD11b^+^Ly6C^+^Ly6G^−^; neutrophils: CD45^hi^CD11b^+^Ly6C^+^Ly6G^+^; other: CD45^hi^CD11b^+^Ly6C^−^Ly6G^−^) and lymphocyte (T cells: CD45^hi^CD11b^−^CD19^−^CD3^+^; B cells: CD45^hi^CD11b^−^CD3^−^CD19^+^MHCII^+^; NK/T cells: CD45^hi^CD11b^−^CD19^−^NKp46^+^DX5^+^; other: CD45^hi^CD11b^−^CD19^−^CD3^−^NKp46^−^DX5^−^) subsets in the ischemic brain (**f** and **g**, respectively). Brain concentrations of TNF-α and IL-1β in sham and stroke mice are shown (**f**; *N* = 4–5/group). CD200R1-positive CD3^+^ T cells in the ischemic brain express a higher percentage of T cell activation markers CD11a (**j**, **k**), CD69 (**l**, **m**), and PD-1 (**n**, **o**) compared to their CD200R1-negative counterparts (*N* = 12/group). FMO controls were used to determine positive gating. Asterisks adjacent to group labels designate an effect of genotype by two-way ANOVA. Error bars show mean SEM. KO knockout, WT wild-type, SEM standard error of mean. *p < 0.05; **p < 0.01; ***p < 0.001

Next, we examined whether the increased behavioral deficits seen in CD200R1-KO mice at day 7 after stroke were associated with persistent neuroinflammation. The immunohistological analysis confirmed a significant increase in Iba1-positive microglia/macrophages in the striatum of CD200R1-KO mice compared to WT controls at 7 days after stroke (Fig. 4a, b; *p* = 0.045). Flow cytometry analysis confirmed that microglia counts were significantly elevated out to day 7 in CD200R1-KO mice (Fig. 4c, d; *p* = 0.039). As the day 7 post-stroke period is characterized by the delayed recruitment of T cells [36–39], we hypothesized that lymphocyte numbers would be more abundant in the brain of CD200R1-KO mice compared to their WT counterparts. An analysis of total CD45^hi^ peripheral leukocyte counts found that CD200R1-KO mice had approximately three times the number of infiltrating cells as controls at 7 days, despite having similar myeloid-to-lymphocyte ratios (Fig. 4e; *p* = 0.0005, *F*(1, 42) = 14.12). Further analysis revealed that Ly6C^+^Ly6G^−^ monocytes made up the majority of infiltrating myeloid cells, whereas T cells (gated by CD3^+^) accounted for the majority of infiltrating lymphocytes in CD200R1-KO mice (Fig. 4f, g, respectively; *p* ≤ 0.001). Interestingly, nearly all myeloid and lymphocyte subsets were present in higher numbers in CD200R1-KO mice after stroke, suggesting that CD200R1 regulates the amplitude of leukocyte infiltration into the ischemic brain. No differences in gross vascular anatomy or hemorrhagic transformation as measured by India ink staining and hemoglobin assay, respectively, were seen between genotypes (Additional file 2: Figure S2). ELISA analysis of TNF-α and IL-1β showed significantly higher concentrations of these pro-inflammatory cytokines in ischemic brain tissue of KO mice compared to WT controls at day 7 after stroke (Fig. 4h, i, respectively; *p* < 0.001). CD200R1-positive T cells co-expressed CD11a, CD69, and PD-1 in greater number and higher levels compared to CD200R1-negative T cells in the ischemic brain, suggesting that CD200R1 serves as a useful T cell activation marker and a potential target for CD200R1 agonist therapy (Fig. 4j–o; *p* = 0.0381, 0.0006, and 0.025, respectively). Together, these findings suggest that at the level of the CNS, CD200R1 signaling inhibits the early transmigration and/or chronic entry of leukocytes into the ischemic brain over time, which is required to facilitate a full recovery.

### The poorer functional outcomes in CD200R1-deficient mice after stroke are associated with profound peripheral immune suppression 1 week after stroke

**Fig. 5:**
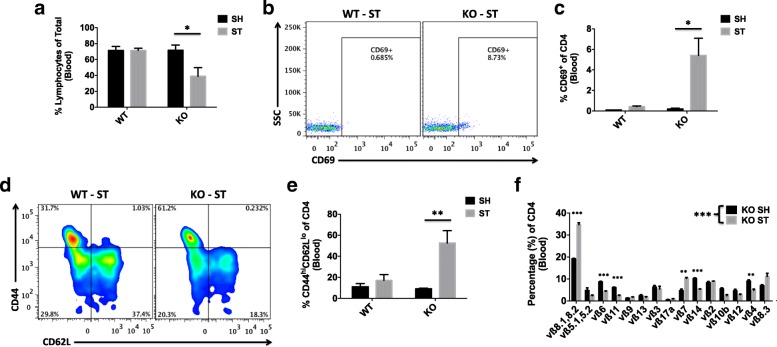
Worsened stroke outcomes in CD200R1-deficient mice are associated with profound peripheral immune suppression 1 week after stroke. The percentage of circulating lymphocytes was significantly lower in CD200R1-knockout mice at 7 days after MCAO compared to wild-type littermate controls (**a**; *N* = 5/group). Stroke-induced lymphopenia was associated with an increase in the percentage of circulating CD4 T cells expressing the early T cell receptor (TCR) activation marker, CD69 (**b**, **c**), and those with effector memory (CD44^hi^CD62L^lo^) phenotype (**d**, **e**). A mouse vβ TCR screening panel containing 15 FITC-conjugated monoclonal antibodies was used to assess TCR vβ usage in CD4 T cells from the blood of CD200R1-deficient mice that underwent sham and stroke surgery. The mean percentage for each of the 15 subfamilies of T cell receptor vβ is displayed (**f**). Significant differences were found between the groups illustrating the narrowing of the CD4 TCR repertoire in CD200R1-knockout mice after MCAO (*N* = 5/group). Cell-specific FMO controls were used to determine positive gating. Asterisks adjacent to group labels designate a significant interaction between groups by two-way ANOVA. Error bars show mean SEM. KO knockout, WT wild-type, SH sham, ST stroke, SEM standard error of mean. *p < 0.05; **p < 0.01; ***p < 0.001

The precipitous functional decline that occurs after day 3 of stroke in surviving CD200R1-KO mice suggests that the peripheral immune state is persistently activated and functionally impaired. We therefore examined circulating blood leukocytes for evidence of continued dysregulation. Consistent with the notion of secondary bacterial infection, CD200R1-KO exhibited prolonged lymphopenia at day 7 after stroke (Fig. 5a). Complete blood count results confirmed that the number of circulating white blood cells was significantly lower in KO mice after stroke (Additional file 3: Figure S3)*.* Circulating CD4 T cells from CD200R1-KO mice showed exaggerated upregulation of the early TCR activation marker, CD69 (Fig. 5b, c). Moreover, a significant increase in the frequency of CD44^hi^CD62l^lo^ effector memory (Tem) cells was found within the CD4 subset (Fig. 5d, e), suggesting active memory conversion of naïve T cells following antigenic stimulation. The gross change observed in the CD4 memory population may indicate expansion and a subsequent narrowing of the TCR repertoire. Indeed, subsequent studies of blood CD4 T cells in CD200R1-KO mice showed a marked re-arrangement in TCRvβ usage at 1 week after stroke, with significantly higher frequency shifts in the subfamily member’s vβ8.1 and 8.2 (Fig. 5f).

### CD200R1-deficient mice develop spontaneous bacterial infection of the lung after stroke

**Fig. 6:**
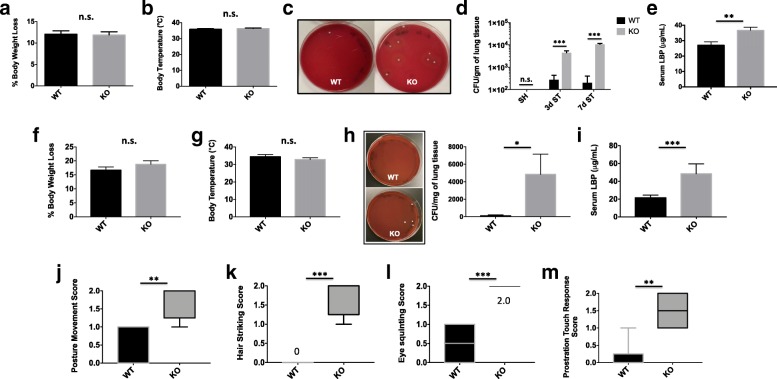
CD200R1-deficient mice exhibit greater spontaneous bacterial colonization of the lung after stroke and enhanced sickness behavior in a LPS model of systemic immune challenge. CD200R1-mediated susceptibility to spontaneous bacterial infection was addressed after MCAO and in a second model of LPS-induced systemic immune challenge. The percentage of body weight loss (**a**) and core body temperature (**b**) is shown at day 3 after stroke is shown (*N* = 9/group). Representative images of bacterial colonies grown from lung homogenates on blood agar plates following acute stroke are shown (**c**). Lung colony-forming units (CFU) counts were statistically higher at days 3 and 7 in injured CD200R1-KO mice compared to CD200R1-WT control littermates (**d**). Serum concentrations of lipopolysaccharide-binding protein (LBP) at day 3 after stroke are shown (**e**). Percentage body weight loss (**f**) and body temperature (**g**) were quantified at day 3 after i.p. LPS injection (*N* = 6/group). Bacterial colonies cultured on blood agar demonstrated statistically higher lung colony-forming units (CFU) counts in injured CD200R1-KO mice compared to CD200R1-WT control littermates after endotoxin challenge (**h**; *N* = 6/group). Serum concentrations of lipopolysaccharide-binding protein (LBP) at day 3 after stroke are shown (**i**). Sickness behaviors were examined in CD200R1-knockout (KO) and wild-type (WT) control littermates 3 days after systemic LPS administration. CD200R1-KO mice had significantly worse sickness scores for postural movement (**j**), hair striking (**k**), eye squinting (**l**), and prostration touch response (**m**). For all behavioral experiments, *N* = 6/group. Error bars show mean SEM. SH sham, ST stroke, KO knockout, WT wild-type, SEM standard error of mean. *p < 0.05; **p < 0.01; ***p < 0.001

Next, we wanted to examine the effects of the augmented lymphopenia in CD200R1 mice on susceptibility to post-stroke infection. Most strikingly, we observed a 52% survival rate for CD200R1-KO mice by day 7 after stroke, significantly lower than was seen for WT controls (83%; *p* = 0.049) (Fig. 1a). No difference in body weight or body temperature was observed during the early stages of stroke (Fig. 6a, b). However, consistent with our earlier results, CD200R1-KO mice showed significantly higher CFUs in lung tissue and increased serum LBP concentrations (Fig. 6c–e). At 72 h after stroke, 37.5% of WT mice and 100% of KO mice had an infection, whereas at day 7, 20% of WT mice and 100% of KO mice had lung infections. These findings suggest that CD200R1 immune inhibitory signaling is important for controlling the spread of bacterial infection in response to ischemic stroke.

### CD200R1-deficient mice exhibit greater spontaneous bacterial colonization of the lung and enhanced sickness behavior after LPS administration

Next, we examined whether these findings could be replicated in systemic endotoxin challenge, a second model of acute neuroinflammation. Despite no difference in body weight loss or body temperature regulation between genotypes (Fig. 6f, g), careful examination revealed increased bacterial colonization of the lung as measured by CFU per milligram tissue and greater serum lipopolysaccharide-binding protein (LBP) concentrations, indicative of bacterial translocation and spontaneous infection (Fig. 6h, i). Infection rates were higher in KO mice after LPS, with 16.6% of WT mice and 100% of KO mice showed lung infection. Three days following LPS injection, CD200R1-KO mice exhibited more severe sickness behaviors including higher severity scores in postural movement, hair striking, eye squinting, and prostration touch responses (Fig. 6j–m). These data suggest that CD200R1-KO mice have a unique sensitivity to systemic inflammatory challenge and that CD200-CD200R1 signaling may be an important regulator of secondary infection control.

## Discussion

In this study, we provide evidence to support the hypothesis that CD200-CD200R1 interactions are important for (1) the peripheral immune control of spontaneous bacterial lung infection post-stroke and (2) downregulating neuroinflammatory responses via (3) attenuation of microglia proliferation and (4) inhibition of monocyte and T cell entry into the ischemic brain and (5) are critical for acute survival and behavioral recovery after stroke. Previous work identified the CD200-CD200R1 signaling axis as a major regulator of microglial homeostasis in the healthy and diseased CNS [5, 40]. Based on this theory, neuronal-microglial interactions impart a unidirectional immune-inhibitory signal that suppresses microglia activation and contribute to the immune-privileged status of the CNS [41]. Our work identifies a novel role for CD200R1 in regulating the systemic response to brain injury, including the entry and function of bone marrow-derived monocytes and T cells within the injured CNS. We show for the first time that CD200R1 inhibitory signaling is critical for preventing spontaneous lung infection, attenuating brain inflammation, improving survival, and promoting functional recovery after stroke. These findings implicate the CD200-CD200R1 immunoregulatory pathway as a novel and powerful therapeutic target in ischemic brain injury.

Mounting evidence highlights the detrimental impact of chronic inflammation on neuroplasticity and long-term recovery following stroke [42]. In this study, we hypothesized that neuroinflammation would be sustained in the absence of CD200R1 inhibitory signaling. Surprisingly, lymphocytes were found to be predominant cell type expressing CD200R1 in the ischemic brain, not microglia, and CD200R1-positive T cells exhibited a higher level of activation than T cells from CD200R1-deficient mice. Mice with higher myeloid-to-lymphocyte ratios in the ischemic brain at 7 days had worse neurological outcomes, potentially implying that enhanced delayed recruitment of lymphocytes and/or augmented resolution of myeloid inflammation facilitates post-stroke recovery. Previous work has shown that CD200R1 activation suppresses macrophage migration in part by downregulating expression of the integrin adhesion molecules CD11a and CD49d [8]. This is consistent with our finding that CD49d is upregulated on circulating CD200R1-deficient monocytes and with earlier studies that have shown increased macrophage numbers in both sterile and autoimmune models of brain injury in CD200-deficient mice. Previous studies have also demonstrated that CD200-KO mice, the ligand for CD200R, show increased blood-brain barrier permeability, greater leukocyte infiltration, and higher pro-inflammatory cytokine levels after LPS administration compared to WT littermate controls [40]. These results suggest that CD200R1 signaling is a powerful regulator of myeloid cell function under sterile inflammatory conditions both at the vascular level (i.e., facilitating entry) and within the injured brain (i.e., modulating function).

To our knowledge, this is the first study to investigate the functional effect of loss of CD200R1 immune-inhibitory signaling in the CNS. The CD200 receptor family has several isoforms, in which all but one (CD200R1) are activating receptors that lack inhibitory motif-bearing proteins. There is an advantage of using transgenic mice containing a genetic deletion of CD200R1 rather than CD200. Any observed effect is a result of the absence of inhibitory signaling rather than a combination of activating and inhibitory functions [43, 44]. The phenotypic similarities in the neuroinflammatory state between CD200- and CD200R1-KO mice imply that the combined functional role of the immune-activating CD200R family members may be negligible [40, 44]. Nonetheless, the potential for CD200 binding to induce both activatory (i.e., CD200R2, CD200R3, CD200R4) and inhibitory (i.e., CD200R1) signaling make matters complicated when trying to determine which pathways are responsible for the phenotype. The off-target effects of CD200R2/3/4 signaling cannot be ruled out when targeting CD200 using genetic knockout, knockdown, Fc drug, or antibody blockade. Genetic deletion of CD200R1, however, selectively removes the inhibitory pathway but leaves the activatory pathways intact. We did not observe CD200R3 expression on adult microglia (not shown); however, its expression after stroke and that of the other activating family members deserves further study. Importantly, given its unique expression on activated or mature tissue-resident myeloid cells and lymphocytes, previous reports indicate no obvious signs of developmental alteration in immune homeostasis, indicating that any limitations due to constitutive loss of CD200R1 during development are minimal [15, 45].

Given the lack of CD200R1 expression on microglia before and after stroke, it is likely that the KO response to injury is primarily mediated by peripheral immune cells. The fact that CD200R1-KO mice exhibit a dramatic susceptibility to infection, only after injury or LPS, highlights the often overlooked importance of these immune signaling pathways in quelling endogenous/exogenous bacterial invasion triggered by brain injury. While it may be counterintuitive to think that increasing the activation level of immune cells (i.e., CD200R1 KO) is detrimental to controlling infection, our results support the conclusion that interfering with the normal balance of activatory versus inhibitory signaling causes a lack of control of the immune response. The uncalibrated immune response and mortality that follows underscore the importance of peripheral immune inhibitory signaling in fighting infection. Although this mechanism is not well understood, it is not without precedent. Rygiel and colleagues elegantly showed that CD200 deficiency enhances pathological T cell responses during influenza infection [46]. CD200-KO mice developed more severe disease and greater lung infiltration, which were completely prevented by depletion of T cells before infection. The peripheral changes in T cell function noted in our study are dramatic, and the high level of CD200R1 expression on these cells, specifically TCR-activated subsets, strongly implicates them in the development of stroke-induced lung infection. The earlier proposed role of hyperactivated microglia might be secondary to the pathological T cell activation [5, 47]. Differences in activation of CD200R1-deficient dendritic cells could also account for these changes.

Recently it was shown that ICV injection of exogenous recombinant CD200 protein at the time of permanent MCAO reduced the expressions of Iba-1, IL-1β, TNF-α, and IL-10 at 48 h after ischemia [48]. The authors reported a negative correlation between decreasing CD200 levels after injury and increasing neuronal death. While this study highlights the therapeutic potential of this pathway in stroke, it did not assess functional outcomes. Our data indicates CD200-CD200R1-mediated regulation of immunity is essential for survival and improved behavioral outcome following stroke. Acute mortality rates from stroke are high, as the majority of severe stroke patients die within the first week of hospitalization [49]. Mirroring the clinical scenario, nearly half of the mice lacking CD200R1 did not survive the first week following a moderate ischemic brain injury, despite no difference in infarct size between the groups. While survivor bias may partially explain the lack of difference in neuronal injury, it does not explain why inflammation was exacerbated and behavioral deficits were worsened. Although behavioral testing was not performed at day 3 to prevent further stress and mortality in the KO group, it is possible that stroke-induced motor impairment occurs much earlier than day 7 in these mice. Our data, however, also suggests a significant role for CD200R1 signaling in the peripheral immune compartment following stroke. Infectious complications, such as post-stroke sepsis and pneumonia, tend to occur within the first week post-stroke and are strong independent risk factors of death [50]. In these experiments, CD200R1-KO mice exhibited progressive weight loss, body temperature dysregulation, peripheral immune dysfunction, and bacterial lung colonization indicative of systemic infection. Indeed, KO mice demonstrated severe and persistent T cell lymphopenia after stroke. Others have also reported that LPS-induced mortality is higher in CD200R1-KO mice versus WT, consistent with our findings [51]*.* Given the role of CD200R1 in regulating T cell tolerance and systemic immune function, it is possible that CD200R1-KO mice are unable to mount the appropriate adaptive responses to mitigate the detrimental effects of spontaneous bacterial infections that are known to occur in the MCAO model [14, 52, 53]. Within the lymphocyte lineage, CD200R1 is primarily expressed on activated memory T and B cells [45], indicating that it may play a critical role in the adaptive immune response to stroke. Indeed, previous work has shown that CD200^−/−^ mice show increased numbers of activated leukocytes and pro-inflammatory cytokines and higher mortality in response to experimental septicemia [54]. Surprisingly, the authors found that CD200 deficiency did not affect bacterial clearance. Differences in injury models and pathology likely account for these discrepant findings*.* Interestingly, previous work has shown that mice lacking CD200 display an enhanced sensitivity to influenza infection, leading to delayed resolution of inflammation and death [55]. These findings are in line with our own and indicate that defects in immune inhibitory signaling in the systemic immune environment (i.e., sepsis) may serve as a critical driver of stroke-related mortality. Interestingly, selective expansion of memory CD4 T cells expressing TCRvβ 8.1/8.2 was found in KO mice after stroke. This subset of murine vβ8^+^ T cells recognizes the superantigen staphylococcal enterotoxin B, a virulent determinant in *Staphylococcus aureus* septicemia [56]. Thus, therapeutic targeting of immune inhibitory axes such as the CD200R1 pathway may prove beneficial in reducing secondary infectious complications in already immunocompromised individuals after stroke.

Past studies have shown ample evidence for a protective role of bone marrow-derived monocytes at acute time points following stroke [57]. These studies highlight the beneficial functions of this early wave of infiltrating monocytes in eliminating necrotic cells, maintaining vascular stability, and inducing M2 polarization in microglia [58]. The role of monocytes in the post-acute or chronic phase of ischemic brain injury is less certain, as any restoration to normal CNS homeostasis likely requires the expedient removal of all non-resident immune cells from the brain. Our findings that CD200R1-KO mice exhibit greater microglia/monocyte numbers, but similar neurological outcomes, at 3 days indicates that the quality of inflammation may outweigh the quantity. Indeed, CD200R1-deficient monocytes from the ischemic brains exhibited greater phagocytic activity and diminished expression of pro-inflammatory cytokines compared to the WT counterparts, suggesting an important role for CD200 in monocyte/macrophage priming and/or polarization in response to ischemic stimuli. However paradoxical, these data are consistent with those reported from mice deficient for CX3CR1^−/−^, an immune regulatory receptor similarly important for maintaining CNS homeostasis, which demonstrates an early protective neuroinflammatory environment after ischemic stroke [59, 60]. Despite this early beneficial phenotype, the inability to properly polarize and clear these cells from the ischemic brain over time appears to have detrimental consequences for survival and neurologic recovery. The functional effect of CD200R1 deletion on monocyte activity appears to be cell-intrinsic rather than secondary to direct neuronal injury. These data strongly implicate infiltrating monocytes as the primary myeloid cells inhibited by the CD200R1 pathway following acute stroke. Furthermore, the worsened behavioral deficits of CD200R1-KO mice appear to be largely driven by the significant increase in delayed CNS monocyte and lymphocyte infiltration, defining a new role for CD200R1 in modulating the migratory activity of peripherally derived leukocytes after injury. Taken together with the dramatically higher levels of pro-inflammatory cytokines found in KO brains at day 7, these data suggest that CD200R1 is required for resolving neuroinflammation following ischemia.

The expression pattern of CD200 and CD200R1 is relevant to its reported role as a homeostatic regulator in the brain and forms the basis of the theory that neuronal-glial interactions are critical for maintaining microglial quiescence [5, 47, 61]. The developmental expression pattern of CD200 in the healthy brain has been examined in detail [62]. CD200 is expressed in cortical neurons throughout life, in astrocytes during early development, and in endothelial cells during adulthood. Luminal expression of CD200 has been reported by others and suggested to be involved in the suppression of monocyte adhesion to the brain endothelium [63]. Temporal and regional changes in CD200 gene and protein expression have also been described in rodents after stroke. CD200 mRNA levels were lower in the ischemic core during the first week after stroke [11]. In a separate study, modest but significant reductions in CD200 protein expression were found in the ischemic hemisphere during the first 48 h [48]. Our study examined CD200 protein levels in the ipsilateral hemisphere at 72 h but did not see any change. This may be explained by previous work which shows CD200 gene expression is dependent on the region of the sampled ischemic brain tissue, with greater transcription occurring in the infarct core from 1 to 2 days and subsequently lower transcription levels from 3 to 5 days relative to peri-infarct and contralateral regions [64]. Interestingly, Masocha showed that gene expression was significantly increased in the brain at 4 h after systemic administration of LPS, returned to baseline by 24 h, but were dramatically lower at 1 year [65]. The long-term impact of stroke on CD200 expression and its contribution to chronic neurodegeneration remains to be seen. Our study has identified activated peripheral immune cells as the predominant CD200R1 expressers in stroke, providing new cellular targets for systemically administered CD200 supplementation therapies.

To date, our knowledge of the CD200-CD200R1 signaling axis in stroke patients is in its infancy. It is not yet known whether CD200R1-KO mice exhibit a threshold effect to ischemic brain injury, as a shorter occlusion time (e.g., 30 min) resulting in smaller infarct volumes, and less inflammatory stimuli were not evaluated in this study. As stroke is a disease that primarily affects the elderly, future studies will be needed to assess the outcomes in aged knockout mice. In addition, the requirement for immune inhibition may be greater with increasing injury and in the context of chronic inflammation, certain contexts such as chronic inflammation. Future studies measuring soluble CD200 levels in the plasma of stroke patients could determine whether this molecule serves as a potential biomarker of neuronal injury or, perhaps, could be used to identify patients at risk for systemic immune suppression and subsequent infections, opening new doors to CD200R1 therapies.

## Conclusion

By using CD200R1-deficient mice, we demonstrate that immune inhibitory signaling is essential for survival following moderate-to-severe stroke. CD200R1 contributes to the resolution of neuroinflammation, uniquely impacts monocyte responses, and is an important regulator of the body’s peripheral immune response to bacterial infection. Our observations provide new insights into the contribution of the CD200-CD200R1 inhibitory pathway to the progression of neuroimmune dysfunction following stroke and highlight a novel role for CD200R1 for the prevention of systemic immunosuppression and secondary infection after stroke. Inhibitory receptors such as CD200R1 evolved as a strategy to prevent autoimmunity and prolonged inflammation in immune-privileged regions like the CNS. The compensatory increase in inhibitory receptor expression on the surface of acutely and chronically activated immune cells implies that this system can be exploited to ameliorate post-stroke inflammation and restore CNS homeostasis. These findings suggest that targeting the CD200-CD200R1 signaling axis may hold therapeutic potential for the prevention of acute infection and the treatment of chronic brain inflammation following stroke.

## Additional files

Additional file 1: **Figure S1.** CD200 protein concentrations in the ischemic hemisphere and plasma 72 h after stroke. ELISA measurement of CD200 protein concentrations in the ischemic brain (a) and plasma (b) 72 h after stroke shows no significant change in wild-type mice (*N* = 5–8/group). Error bars show mean SEM. Abbreviations: SH sham, ST stroke, SEM standard error of mean. (PDF 37 kb)

Additional file 2: **Figure S2.** No differences in gross vascular anatomy or in hemorrhagic transformation between WT and KO mice 7 days after stroke. Representative images of India ink-stained brains show no overt difference in large vessel anatomy between genotypes under normal conditions (a; ventral view). Hemoglobin concentrations in the brain 7 days after stroke show no difference in hemorrhagic transformation between genotypes (b; *N* = 5/group). Error bars show mean SEM. Abbreviations: WT wild-type, KO knockout, SEM standard error of mean. (PDF 55 kb)

Additional file 3: **Figure S3.** Complete blood counts demonstrate significant leukopenia in KO mice 7 days after stroke. Blood leukocyte counts show similar values between genotypes after sham surgery. Leukopenia and lymphopenia are exacerbated in KO, but not WT mice, 7 days after stroke (*N* = 4–5/group). Error bars show mean SEM. Abbreviations: SH sham, ST stroke, WT wild-type, KO knockout, SEM standard error of mean. (PFD 96 kb)

## Abbreviations

CFU: Colony-forming unit
CNS: Central nervous system
CV: Cresyl violet
DAPI: 4′,6-Diamidino-2-phenylindole, dihydrochloride
DMSO: Dimethyl sulfoxide
FACS: Fluorescence-activated cell sorting
FMO: Fluorescence minus one
i.p.: Intraperitoneal
KO: Knockout
LBP: Lipopolysaccharide-binding protein
LPS: Lipopolysaccharide
PBS: Phosphate-buffered saline
PFA: Paraformaldehyde
RPM: Rotations per minute
RPMI: Roswell Park Memorial Institute medium
TCR: T cell receptor
tMCAO: Transient middle cerebral artery occlusion
TTC: 2,3,5-Triphenyltetrazolium chloride
WT: Wild-type
YG: Yellow green
