# Supplementary figures and images for: CD200-CD200R1 inhibitory signaling prevents spontaneous bacterial infection and promotes resolution of neuroinflammation and recovery after stroke
Source: J Neuroinflammation. 2019 Feb 18;16:40. doi: 10.1186/s12974-019-1426-3 (PMC6378746; doi:10.1186/s12974-019-1426-3)

a)

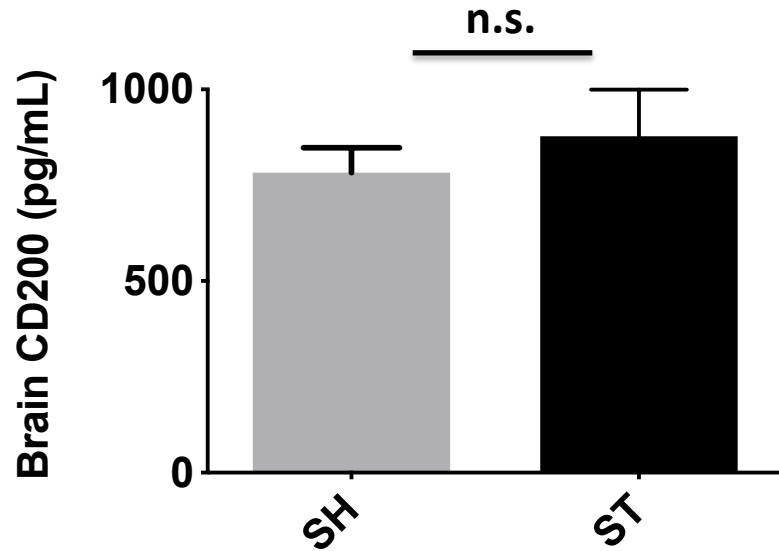

b)

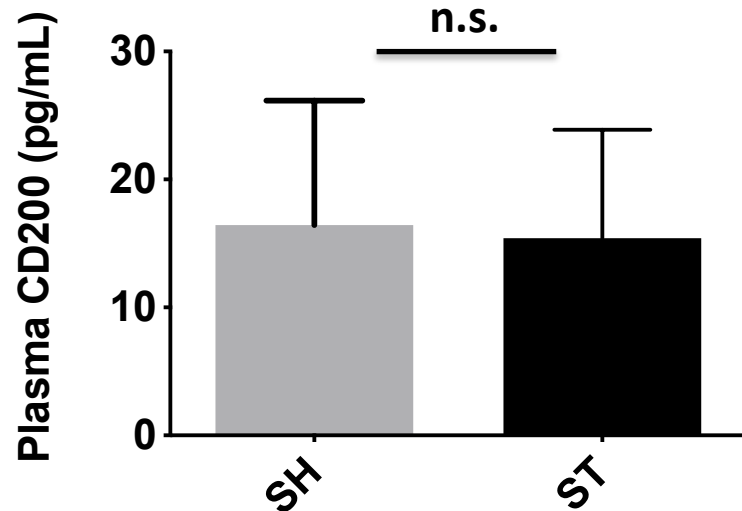

Supplement: Supplementary file 1 — Figure S1. CD200 protein concentrations in the ischemic hemisphere and plasma 72 h after stroke. ELISA measurement of CD200 protein concentrations in the ischemic brain (a) and plasma (b) 72 h after stroke shows no significant change in wild-type mice (N = 5–8/group). Error bars show mean SEM. Abbreviations: SH sham, ST stroke, SEM standard error of mean. (PDF 37 kb) [file 12974_2019_1426_MOESM1_ESM.pdf]

a)

WT

KO

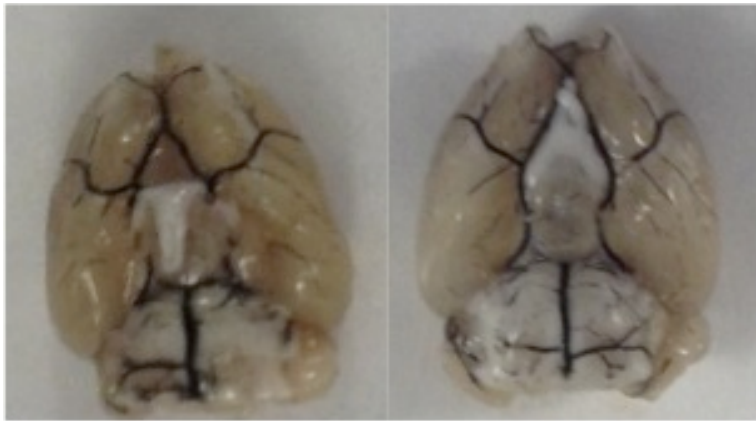

b)

Brain Hemoglobin  
(pg/mg protein)

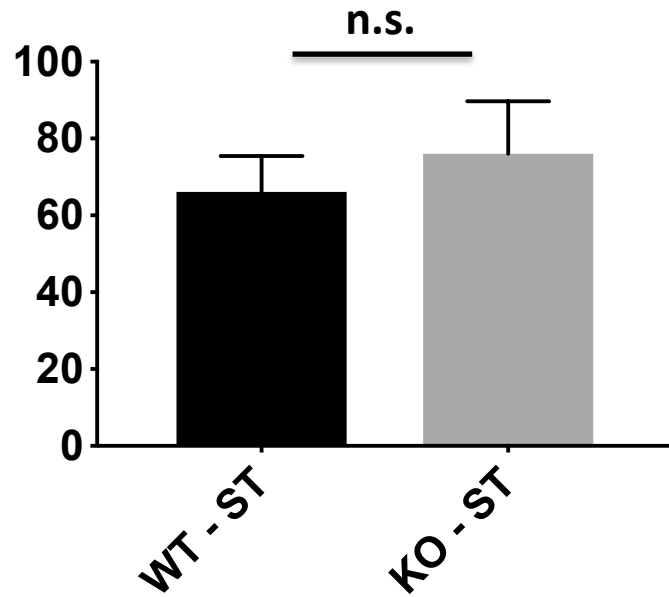

Supplement: Supplementary file 2 — Figure S2. No differences in gross vascular anatomy or in hemorrhagic transformation between WT and KO mice 7 days after stroke. Representative images of India ink-stained brains show no overt difference in large vessel anatomy between genotypes under normal conditions (a; ventral view). Hemoglobin concentrations in the brain 7 days after stroke show no difference in hemorrhagic transformation between genotypes (b; N = 5/group). Error bars show mean SEM. Abbreviations: WT wild-type, KO knockout, SEM standard error of mean. (PDF 55 kb) [file 12974_2019_1426_MOESM2_ESM.pdf]
