# Supplementary material for: CD200-CD200R1 inhibitory signaling prevents spontaneous bacterial infection and promotes resolution of neuroinflammation and recovery after stroke
Source: J Neuroinflammation. 2019 Feb 18;16:40. doi: 10.1186/s12974-019-1426-3 (PMC6378746; doi:10.1186/s12974-019-1426-3)
Supplement: Supplementary file 3 — Figure S3. Complete blood counts demonstrate significant leukopenia in KO mice 7 days after stroke. Blood leukocyte counts show similar values between genotypes after sham surgery. Leukopenia and lymphopenia are exacerbated in KO, but not WT mice, 7 days after stroke (N = 4–5/group). Error bars show mean SEM. Abbreviations: SH sham, ST stroke, WT wild-type, KO knockout, SEM standard error of mean. (PFD 96 kb) [file 12974_2019_1426_MOESM3_ESM.pdf]

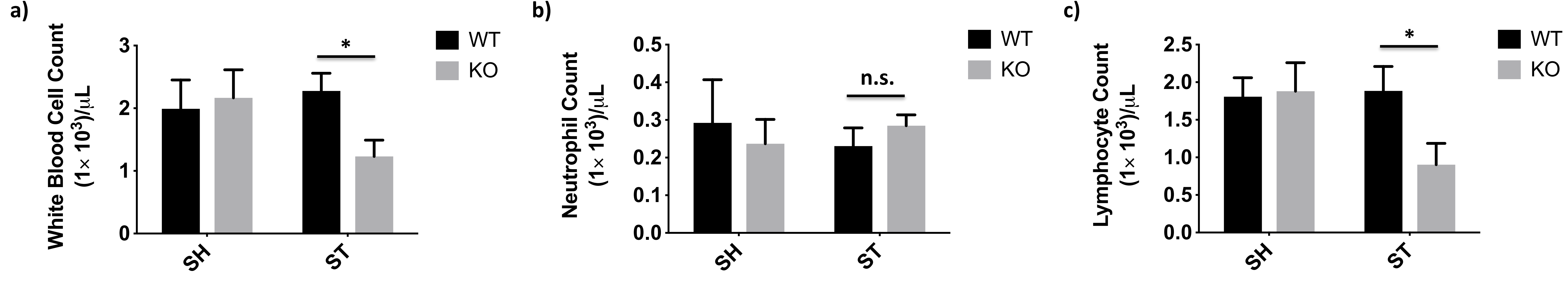
a)
White Blood Cell Count
(1× 10³)/μL
3
2
1
0
SH
ST
*
WT
KO
b)
Neutrophil Count
(1× 10³)/μL
0.5
0.4
0.3
0.2
0.1
0.0
SH
ST
n.s.
WT
KO
c)
Lymphocyte Count
(1× 10³)/μL
2.5
2.0
1.5
1.0
0.5
0.0
SH
ST
*
WT
KO
